# VISTA+/CD8+ status correlates with favorable prognosis in Epithelial ovarian cancer

**DOI:** 10.1371/journal.pone.0278849

**Published:** 2023-03-23

**Authors:** Aida Jlassi, Maroua Manai, Maram Morjen, Ghada Sahraoui, Monia Elasmi Allal, Ines ELBini-Dhouib, Lamia Naija, Lamia Charfi, Rim Rejaibi, Melika Ben Ahmed, Naziha Marrakchi, Najet Srairi-Abid, Amel Mezlini, Mohamed Manai, Karima Mrad, Raoudha Doghri

**Affiliations:** 1 Department of Biology, Mycology, Pathologies and Biomarkers Laboratory (LR16ES05), Faculty of Sciences of Tunis, University of Tunis El Manar, Ariana, Tunisia; 2 Research Laboratory of Precision Medicine/Personalized Medicine and Oncology Investigation (LR21SP01), Tunis, Tunisia; 3 Human Genetics Laboratory (LR99ES10), Faculty of Medicine of Tunis, University of Tunis, El Manar, Tunis, Tunisia; 4 Department of Medicine, Division of Hematology-oncology, New York, New York, United States of America; 5 Laboratory of Biomolecules, Venoms and Theranostic Applications (LR20IPT01), Pasteur Institute of Tunis, University of Tunis, El Manar, Tunis, Tunisia; 6 Department of Pathology, Salah Azaiez Institute, Tunis, Tunisia; 7 Faculty of Medicine of Tunis, University of Tunis, El Manar, Tunis, Tunisia; 8 Biochemistry Laboratory, La Rabta Hospital, Tunis, Tunisia; 9 Department of Surgical Oncology, Salah Aziz Institute, Tunis, Tunisia; 10 Laboratory of Transmission, Control and Immunobiology of Infections - LR16IPT02, Pasteur Institute of Tunis, University of Tunis, El Manar, Tunis, Tunisia; 11 Department of Medical Oncology, Salah Aziz Institute, Tunis, Tunisia; University of Turin, S. Anna Hospital, ITALY

## Abstract

Immunotherapy by blocking immune checkpoint regulators has emerged as a new targeted therapy for some cancers. Among them V-domain Ig suppressor of Tcell activation (VISTA) which is identified as a novel checkpoint regulator in ovarian cancer. This study aimed to investigate the VISTA role in Epithelial ovarian cancer (EOC), and its relationship with tumor-infiltrating lymphocytes (TILs) markers and its prognostic value. The expression of VISTA, CD3, CD8, CD4, FOXP3, and CD56 was assessed in 168 EOC tissue microarrays (TMA) by immunohistochemistry (IHC). In addition, associations between VISTA, TILs, clinicopathological variables, and overall survival (OS) were analyzed. VISTA expression in IGRov1 cells, as well as in PBMC of EOC patient, was evaluated by western blot. VISTA expression was detected in 64,28% of tissues, among which 42.3% were positive for tumor cells (TCs), and 47,9% were positive for immune cells (ICs). In univariate analysis, VISTA expression was significantly associated with a high density of TILs:CD3+ (*p =* 0,001), CD4+ (*p =* 0,002) and CD8+ (*p*≤0,001), in ICs but not in TCs. In terms of OS, multivariate analysis showed a significant association between the high density of CD8+ TILs and VISTA positive staining in ICs (*p* = 0,044), but not in TCs (*p* = 0,108). Kaplan-Meier curves demonstrated no correlation between VISTA expression and prolonged OS in both ICs (*p* = 0,841) and TCs (*p* = 0,090). Classification of EOC tumor microenvironment based on VISTA and CD8+TILs expression, demonstrated four immune subtypes: VISTA+/CD8+, VISTA+/CD8-, VISTA-/CD8+ and VISTA-/CD8-. The dual positive VISTA+/CD8+ subtype was significantly associated with prolonged OS in both TCs and ICs (*p* = 0,012 and *p*≤0,01, respectively), whereas patients with VISTA+/CD8- had the worst OS. Our results showed that VISTA is highly expressed in the IGRov1 cell line and LT-CD8 from a patient with EOC. Our results highlighted the association of VISTA expression and CD8+ TILs in EOC, with prolonged OS in patients with VISTA+/CD8+ and proposed VISTA as a potential immunotherapeutic target in EOC.

## Introduction

Ovarian cancer is the leading cause of gynecological cancer-related mortality worldwide, with more than 90% in Epithelial ovarian cancer (EOC). More than 75% of patients are diagnosed at an advanced stage [1] with more than 70% chemoresistance [2]. Consequently, ovarian cancer disseminates and has frequent relapses, with poor prognosis. Immunotherapy strategies for ovarian cancer are still being developed and tested in numerous clinical trials [3]. Immunotherapy, by blocking the immune checkpoint regulators programmed death ligand (PDL1) and cytotoxic T lymphocyte antigen 4 (CTLA4), has emerged as a new target therapy for some cancer, with significant long-term clinical results [4]. V-domain Ig suppressor of T cell activation (VISTA) is a recently discovered inhibitory immune checkpoint molecule belonging to the B7 family and shares sequence homology with PD-1 and PD-L1 [3]. VISTA encoded by the *c10orf54* gene, plays a potential function as a co-inhibitory ligand for T cells. It inhibits T-cell activation, proliferation and the production of cytokines, such as interleukin-2 (IL-2), IL-10, TNFα, and IFNγ secreted by CD4 and CD8 Tcells. In addition, high expression of VISTA protein enhances the conversion of naïve T cells into Treg [1].

VISTA was also reported as a regulator of anti-tumor immunity [5], and its expression in tumor cells inhibits the function of T lymphocytes [2]. VISTA expression is heightened within the tumor microenvironment (TME), and its blockade enhances the TME anti-tumor immune responses in mice [6]. VISTA is primarily expressed in hematopoietic cells (myeloid cells, macrophages and lymphoid cells) and only a few studies have evaluated its expression in cancer cells [7–9].

Notably, VISTA expression is associated with ovarian cancer metastases [10], but its role in OC development was not yet investigated.

Thus, in the current study we aimed to investigate the VISTA expression and its relationship with CD8+TILs, as a significant effector of adaptative immunity, with the Natural Killer (NK) as an effector of innate immunity and clinical outcomes in ovarian carcinoma, to understand the role and status of this novel checkpoint in ovarian carcinoma microenvironment and to check whether its targeting enhances anti-tumor response for EOC immunotherapy.

## Materials and methods

EOC-TMA specimens were collected from Salah Azaiez Institute, and all patients provided specimens with written informed consent and approval from the Institutional Ethics committee at Salah Azaiez Institute (N°2150, September 2020) were obtained in this study. All methods were performed following with the relevant guidelines and regulations. Our study has been conformed with the Code of Ethics of the World Medical Association (Declaration of Helsinki), printed in the British Medical Journal (July 18 ^th^1964).

### Clinicopathological study

#### Patients and samples

The immunohistochemical staining for VISTA expression was performed on a tissue microarray (TMA) of 171 cores belonging to 171 patients with (EOC) collected between 2000 and 2017 at Salah Azaiz Institute (ISA) of Tunis, Tunisia.

The tissues were obtained from the Pathology Department in ISA. It were formaldehyde-fixed and paraffin-embedded. Available clinicopathological data and signed informed patient consent were collected. The institutional ethics committee approved this study at ISA.

#### Tissue Microarrays (TMA) construction

All samples were spotted onto a TMA before IHC analysis. One TMA was available for all 171 cases. For each sample, two representative areas were carefully selected from a hematoxylin-eosin-stained section of the donor block. The TMA core size of 1mm from the designated areas was embedded in the recipient paraffin blockusing a specific arraying device (Alphelys, Plaisir, France). Sections were cut and used for Immunohistochemistry (IHC).

#### Immunohistochemistry

First, we validated VISTA antibody cytoplasmic and /or membranous, by using human placenta tissue as a positive control [11]. We performed the IHC on 4-μm paraffin sections pretreated in PT Link at PH6 (Dako Cytomation, Copenhagen, Denmark). VISTA staining was assessed using a monoclonal antibody, anti-VISTA (anti-rabbit, ab257314, Abcam) at 1:100 dilution, to detect the expression of VISTA in both tumoral (TCs) and immune cells (ICs). Tumor-infiltrated cells were defined as dendritic cells, myeloid cells, macrophages, and lymphocytes (direct contact with the tumor cells). VISTA-positive tumor-infiltrating ICs were typically seen as variably sized aggregates towards the periphery of the tumor mass and in stromal bands dissecting the tumor mass. Lymphocytes within the epithelial compartment were not counted. For stratification and statistical analysis, VISTA expression was evaluated with binary positive /negative scoring: VISTA was defined as positive if any staining was visible in the TCs of each sample. VISTA-positive tumor cells (TCs) were calculated by summing the number of VISTA-stained cells (TCs), dividing the result by the total number of viable TCs, and multiplying the quotient by 100.

VISTA–positive immune cells (ICs) were considered as the percentage of tumor-infiltrating ICs (including dendritic cells, macrophages and lymphocytes) in the tumor mass periphery and the stromal bands dissecting the tumor mass at any intensity. The percentage was calculated by summing the number of VISTA-stained ICs divided by the total number of ICs and multiplying the quotient by 100. Two experienced ovarian pathologists (RD and GS) independently analyzed the stained slides using light microscopy. Positive scores were defined as staining in at least 1% of cells (cutoff value), and a negative score was defined as staining in <1% of cells, which was consistent with the cutoff values recommended in previous studies [6].

In addition, the percentage of CD3+, CD4+, CD8+, FOXP3+, and CD56+ lymphocytes compared with that of the nucleated cells in intra-tumoral compartments were assessed. Tumor cores of each case from both tissue microarrays were scored independently, and the average score was used.

#### Tumor-infiltrating lymphocytes (TILs) markers

TILs were evaluated using labeling by the following mouse monoclonal antibodies: CD8 (NCL-L-CD8 clone 4B11, Novocastra) diluted at 1:50, pH9, CD3 (NCL-L-CD3-565, clone LN10, Novocastra) diluted at 1:500 pH6, CD4 (NCL-L-CD4-368, clone 4B12, Novocastra), diluted at 1:100, pH9, CD56 (CD56, FAD191,66556, Novocastra), and FOXP3 (ab20034, clone 236A(E7), Bioscience) diluted at 1:400. The percentage of CD8+, CD3+, CD4+, CD56+, and FOXP3+ lymphocytes compared with that of the nucleated cells in the stromal and tumoral compartments were assessed.

The proportion of these TILs was dichotomized into ‘low’ and ‘high’ groups based on staining scores (corresponding to the median value) of 3%, 1%, 3%, 1% and 1% respectively, for each core, according to the degree of cell densities. A total of 168 samples were included in this analysis after excluding not found samples.

### *Invitro* study

#### Cell culture

Human EOC cell line IGRov1 provided by Pr. José LUIS (CNRS-UMR 7051, Institut de Neuro Physiopathologie (INP), Aix-Marseille Université) obtained from American Type Culture Collection (ATCC, Manassas, Virginia). Cells were routinely maintained at 37°C and 5% CO2 in RPMI medium supplemented with 10% fetal calf serum (FCS). IGRov1 cells were seeded at 1×10^6^ in a 75mm^2^ flask and cultured for 24 hours (h) in RPMI media. Then, cells were trypsinized, washed and mixed with media for immunocytochemistry (ICC) assay.

On the other hand, PBMCs (peripheral blood mononuclear cells) from a patient with EOC were isolated by centrifugation in Ficoll-Histopaque Plus (Amersham Biosciences) from peripheral blood and washed twice with PBS to fully remove non-adherent cells.

LT-CD8 cells in the peripheral blood from patients with EOC were sorted by the CD8+ Cell Biotin-Antibody cocktail human kit (Miltenyi Biotec, Germany), using manual magnetic labeling. Then, LT-CD8 cells were cultivated in RPMI medium supplemented with 10% FCS and containing 20 ng/ml of IL2 to maintain the proliferation of T cells.

#### Protein electrophoresis and Western Blot (WB) analysis

The extracted proteins from LT-CD8 cell lysates were loaded (at 30 μg/ lane), and separated by SDS-PAGE gel-electrophoresis. The separated proteins were electrophoretically transferred on PVDF (polyvinylidene difluoride, Thermo Fisher Scientific, Waham, MA, USA) membrane, using a transfer system (Bio-Rad). First, the membrane was incubated with a blocking solution (5% non-fat milk) for 1 hour (h) at room tempreture (RT) and then incubated overnight with an anti-VISTA primary antibody (1:1000 dilution, anti-rabbit, Abcam). Next, the membrane was washed three times with a PBST solution and incubated with horseradish-peroxidase-conjugated secondary antibodies for 1h. Finally, the membrane was washed 5 times and revealed with enhanced chemiluminescence (ECL). Western Blotting Detection Reagent (Amersham Pharmacia Biotech, Piscataway, NJ, USA).

### Immunocytochemistry (ICC)

IGRov1 cells were washed with distilled water with 10 minutes of centrifugation (3000g), and cells were mixed with PBS. Then, cells were spread on the slides and dry-fixed. After that, all the IHC steps were similarly done (endogenous peroxidase inhibition, primary antibody then secondary antibody incubation, DAB revelation, HE counterstaining) except the antigenic retrieval.

### Statistical analyses

Statistical analyses were performed using (SPSS version 18.0) and GraphPad Prism (version 9). Numbers and percentages were summarized for categorical variables, and median and range for continuous variables. Chi-square tests were used to analyze the correlation between vista protein expression, clinical pathological variables, and TILs (CD3+, CD4+, CD8+, CD65+, and FOXP3). All statistics were 2-sided and the statistical significance was *p*≤0,05. The survival curves were estimated by the Kaplan -Meir method and were compared using the log-rank test. The multivariate Cox regression analysis investigated the relationship between correlative factors and VISTA expression. Uni- and multivariate prognostic analysis for OS were also performed.

## Results

### Clinicopathological parameters and VISTA expression in patients with EOC

Among 168 samples, 146 were High-grade serous ovarian carcinoma (HGSOC), 5 were endometrioid, 3 were mucinous ovarian carcinoma, 10 were ovarian clear cell carcinoma, and 4 were mixed ovarian carcinoma (Table 1).

**Table 1:** Clinicopathological correlations of VISTA expression in 168 EOC patients.

		VISTA in all cells			VISTA in TCs			VISTA in ICs		
Features	Total (N)	Positive (N, %)	Negative (N, %)	*P*	Positive (N, %)	Negative (N, %)	*P*	Positive (N, %)	Negative (N, %)	*P*
**VISTA+**	168	108 (64,28%)	60(35,71%)	**<0,0001** *	71(42,3%)	97(56,7%)	0,092	81(47,9%)	87(52,1%)	**0,026** *
**Age years (median)**	168	56,120±11,75	56,400±11,30	0,881	56,39±11,64	56,09±11,55	0,868	56,45±11,72	55,81±11,39	0,724
**<40 years**	14	10(71,4%)	4(28,6%)	0,837	6(42,9%)	8(57,1%)	0,723	7(50,0%)	7(50,0%)	0,879
**40–60 years**	89	57(64%)	32(36%)		40(44,9%)	49(55,1%)		41(46,1%)	48(53,9%)	
**>60 years**	65	41(63,1%)	24(36,9%)		25(38,5%)	40(61,5%)		32(50,0%)	32(50,0%)	
**FIGO Stage**	168			0,362			0,191			0,457
**I**	16	8(50%))	8(50%)		6(37,5%)	10(62,5%)		6(37,5%)	10(62,5%)	
**II**	17	12(70,6%)	5(29,4%)		6(35,3%)	11(64,7%)		10(58,8%)	7(41.2%)	
**III**	122	78(63,9%)	44(36,1%)		50(41,0%)	72(59,0%)		57(47,1%)	64(52,9%)	
**IV**	11	9(81,8%)	2(18,2%)		8(72,7%)	3(27,3%)		7(63,6%)	4(36,4%)	
**Distant metastasis**	168			0,257			0,073			0,167
**Yes**	16	12(75,0%)	4(25,0%)		10(62,5%)	6(37,5%)		10(62,5%)	6(37,5%)	
**No**	152	96(63,2%)	56(36,8%)		61(40,1%)	91(59,9%)		70(46.4%)	81(53,6%)	
**Pathological type**	168			0,501			0,339			0,341
**High- grade serous carcinoma**	146	95 (65,1%)	51 (34,9%)		62(42,5%)	84(57,5%)		71(49,0%)	74 (51,0%)	
**Endometrioid**	5	4(80%)	1(20%)		3(60%)	2(40%)		1(20%)	4(80%)	
**Mucinous**	3	2(66,7%)	1(33,3%)		1(33,3%)	2(66,7%)		2(66,7%)	1(33,3%)	
**Clear cell**	10	4(40%)	6(60%)		2(20%)	8(80%)		3(30%)	7(70%)	
**Mixed**	4	3(75%)	1(25%)		3(75%)	1(25%)		3(75,0%)	1(25,0%)	
**Chemotherapy(neo)**	117	21(65,62%)	11(34,38%)	0.553	16(50%)	16(50%)	0.380	15(46,87%)	17(53,13%)	0,555
**Yes**	32									
**No**	85	55(64,70%)	30(35,30%)		38(44,70%)	47(55,30%)		40(47,62%)	44(52,38%)	

FIGO: International Federation of Gynecology and Obstetrics. EOC: Epithelial ovarian cancer, VISTA: V-domain Ig suppressor of T cell activation, TCs: Tumor cells, ICs: Immune cells,

*The values had statistically significant differences.

**Fig 1:**
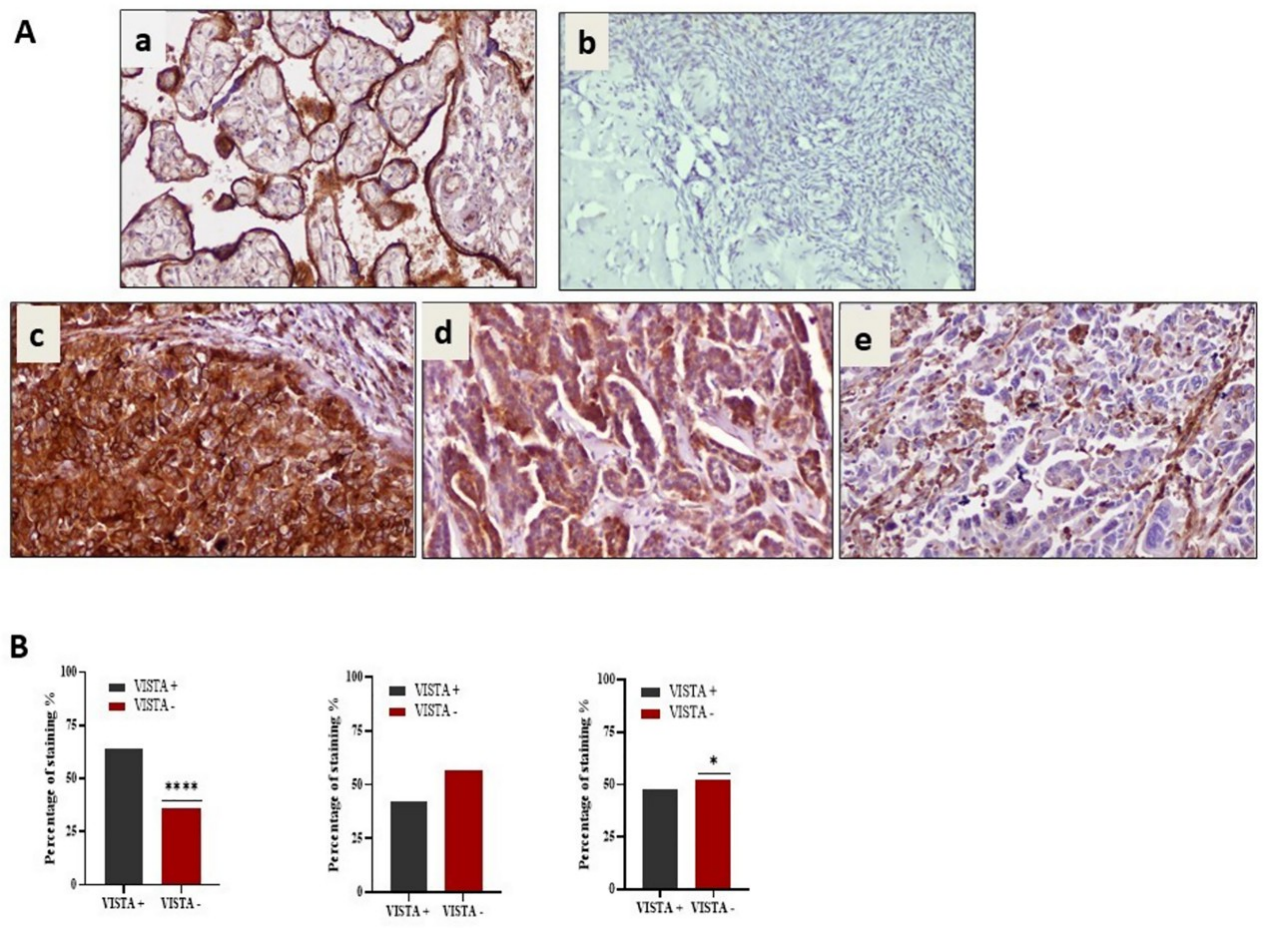
VISTA immunostaining in Epithelial ovarian cancer (EOC). (A)VISTA protein expression in human placenta (positive control) (a), normal ovarian tissue (negative control) (b), VISTA+ in all cells (c), VISTA+ in TCs (d) and VISTA+ in ICs (e) (x200). Positive VISTA staining was cytoplasmic and membranous using a cutoff of 1%. (B) The graphs representing the corresponding percentages of VISTA-positive and negative samples from patients with EOC for each of the three groups (VISTA+ in all cells, VISTA+ in TCs, and VISTA+ in ICs). * for *p*≤0,05 and ****p≤ 0,0001.

According to the International Federation of Gynecology and Obstetrics stage (FIGO) categorization,16 (9,6%) samples were in stage I, 17 (10,2%), in stage II, 122 (73,5%) in stage III and 11 (6,6%) in stage IV. Among the 168 samples, VISTA expression was detected in 108 (64,2%) samples of EOC. VISTA was expressed in 42,3% of TCs, and 47,9% of ICs (Fig 1).

VISTA expression was detected in 65,1% of High- grade serous carcinoma (HGSC) (95/146), 80% of endometrioid ovarian carcinoma (4/5), 66,7% of mucinous carcinoma (2/3), 40% of clear cell carcinomas (4/10) and in 75% of mixed ovarian carcinoma (3/4). VISTA expression tended to be elevated in higher stage tumors, distant metastasis and High-grade serous carcinoma subtype. However, the associations were not statistically significant for the FIGO stage (*p* = 0,362), distant metastasis (*p* = 0,257) and histological subtype (*p* = 0,501).

Table 1 presented VISTA protein expression according to patient characteristics and pathological tumor features.

### Correlation of VISTA expression with the tumor microenvironment and prognostic in patients with EOC

**Fig 2:**
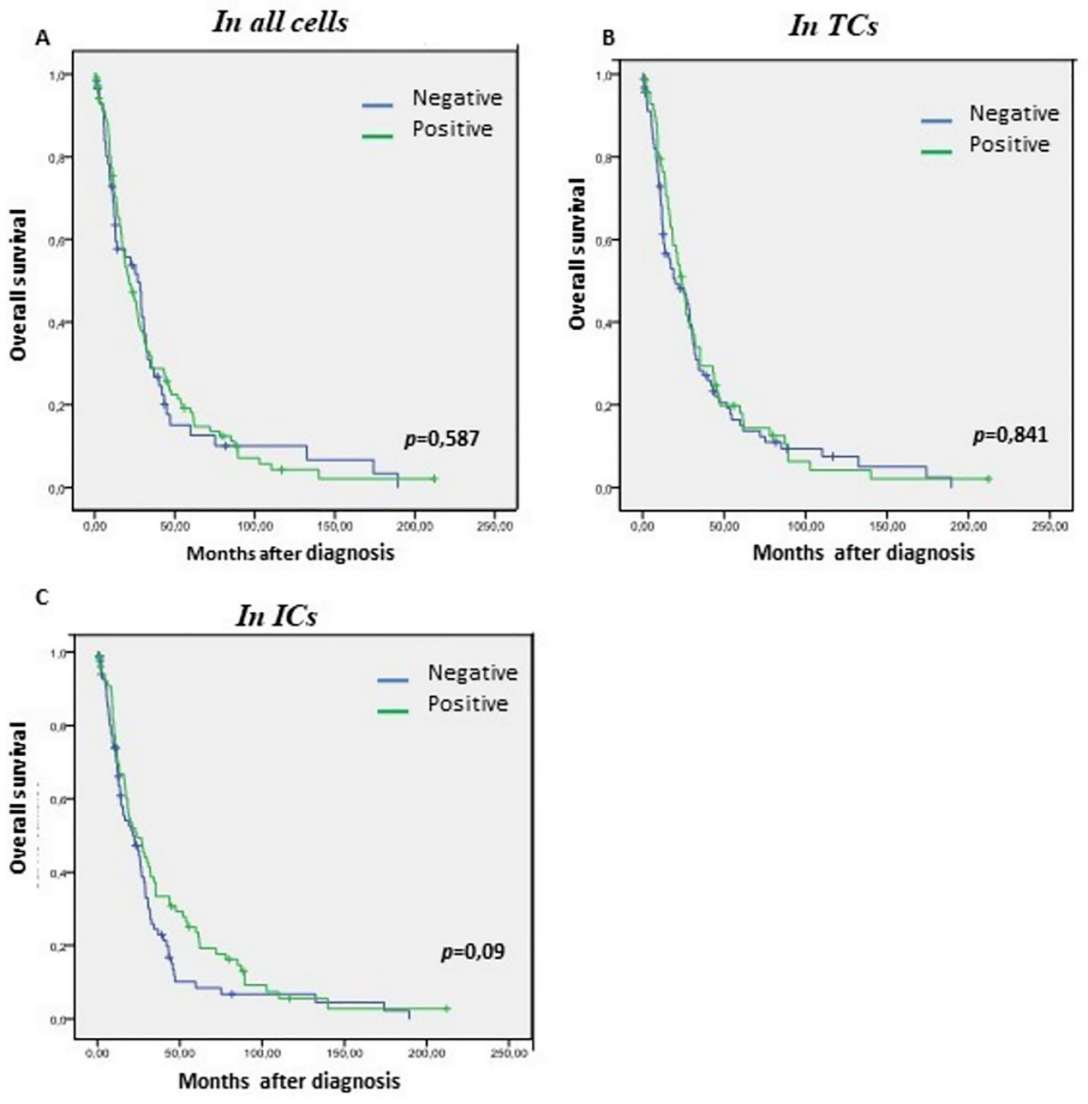
Kaplan-Meier curves showing overall survival (OS) of patients with EOC based on VISTA status. (A) Kaplan-Meier curves of OS in EOC according to VISTA protein expression in all cells. (B) same as figure (A) but in tumor cells (TCs), and (C) same as figures (A) and (B) but in immune cells (ICs). VISTA, V-domain Ig suppressor of T cell activation. The *p*-values of the log-rank test are indicated in the figures.

In patients with EOC, no correlation between VISTA expression and OS, was noticed neither in TCs (*p* = 0,841) nor in ICs (*p* = 0, 090) (Fig 2).

The correlations analysis between VISTA-positive cell types (TCs/ICs), CD4+, CD3+, CD8+FOXP3+ and CD56+TILs showed that VISTA expression in ICs was significantly associated with a high density of CD3+TILs (*p* = 0,011), CD4+ TILs (*p*≤0,001), FOXP3+TILs (*p* = 0,025), and CD8+ TILs (*p*≤0,001). While in TCs, VISTA staining was significantly correlated only with FOXP3 TILs (*p* = 0,057) and CD8+TILs (*p* = 0,029) (Table 2). There is no relationship between NK (CD56+) and VISTA expression, In Univariate analysis, VISTA expression in ICs was significantly associated with high density TILs: CD3+ *(p* = 0,01), CD4+ (*p* = 0,002), Foxp3+ (*p* = 0,007), and CD8+ (*p*≤0,001), but not in TCs.

**Table 2:** Association between VISTA expression and TILs of the TME in patients with EOC.

		VISTA in all cells			VISTA in TCs			VISTA in ICs		
Features	Total (N)	Positive (N, %)	Negative (N, %)	*P*	Positive (N, %)	Negative (N, %)	*P*	Positive (N, %)	Negative (N, %)	*P*
**CD4+ TILs**	142									
**Low (0%)**	51	27(52,9%)	24(47,1%)	**0,001** *	20(39,2%)	31(60,8%)	0,762	16(32,0%)	34(68,0%)	**<0,0001** *
**High (≥1%)**	91	69(75,8%)	22(24,2%)		41(45,1%)	50(54,9%)		57(62,6%)	34(37,4%)	
**CD3+TILs**	158									
**Low (<3%)**	61	37(60,7%)	24(39,3%)	**0,014** *	23(37,7%)	38(62,3%)	0,281	25(41,7%)	35(58,3%)	**0,011** *
**High (≥3%)**	97	64(66,0%)	33(34,0%)		43(44,3%)	54(55,7%)		49(50,5%)	48(49,5%)	
**CD8+ TILs**	126			**<0,0001** *			**0,029** *			**<0,0001** *
**Low (<3%)**	62	31(50,0%)	31(50,0%)		20(32,3%)	42(67,7%)		19(31,1%)	42(68,9%)	
**High (≥3%)**	81	64(79,0%)	17(21,0%)		40(49,4%)	41(50,6%)		52(64,2%)	29(35,8%)	
**FOXP3**:	129									
**High>1**	37	29(78,4%)	8(21,6%)	0,104	20(54,1%)	17(45,9%)	0,057	25(67,6%)	12(32,4%)	**0,025**
**Low (0)**	92	60(65,2%)	32(34,8%)		34(37,0%)	58(63,0%)		43(46,7%)	49(53,3%)	
**CD56**:	144									
**High>1**	7	4(57,1%)	3(42,9%)	0,380	4(57,1%)	3(42,9%)	0,363	2(28,6%)	5(71,4%)	0,178
**Low (0)**	137	95(69,3%)	42(30,7%)		59(43,1%)	78(56,9%)		74(54,0%)	63(46,0%)	

TILs: Tumor Infiltrating Lymphocytes, TME: Tumor microenvironment, TCs: Tumor cells, ICs: Immune cells,

* the values had statistically significant differences (*p*≤0,05).

Multivariate analysis, showed a significant association between VISTA-positive staining in ICs and high density of CD8+ TILs (*p* = 0,044), but not in TCs (*p* = 0,108) (Table 3).

**Table 3:** Univariate and multivariate analysis of prognostic parameters in 168 EOC patients.

	TC				IC			
Variables	Univariate		Multivariate		Univariate		Multivariate	
	*P*	HR (95%CI)	*P*	HR (95%CI)	*P*	HR (95%CI)	*P*	HR (95%CI)
Distant metastasis	0,093	2,486(0,859–7,197)	0,314	2,134(0,488–9,330)	0,225	1,929(0,667–5,575)	0,147	3,231(0,663–15,735)
FIGO stage	0,185	1,361(0,863–2,147)	0,877	0,955(0,534–1,710)	0,454	1,180(0,765–1,822)	0,899	0,961(0,518–1,783)
CD8+TILs	0,088	1,065(0,991–1,145)	0,108	1,091(0,981–1,213)	**<0,0001** *	1,238(1,111–1,378)	**0,044** *	1,167(1,004–1,356)
CD4+TILs	0,63	0,972(0,868–1,090)	0,213	0,891(0,743–1,068)	**0,002** *	1,319(1,106–1,573)	0,173	1,116(0,953–1,307)
CD3+TILs	0,334	1,054(0,947–1,173)	0,658	0,963(0,813–1,140)	**0,010** *	1,186(1,042–1,348)	0,481	1,074(0,880–1,311)
CD56+TILS	0,866	1,027(0,754–1,399)	0,861	0,968(0,676–1,387)	0,212	0,815(0,591–1,124)	0,274	0,803(0,541–1,190)
FOXP3+TILs	0,083	1,189(0,978–1,447)	0,481	1,100(0,844–1,434)	**0,007** *	1,337(1,081–1,654)	0,854	0,973(0,731–1,297)

TILs: tumor-Infiltrating lymphocytes, TCs: Tumor cells, ICs: Immune cells, HR: hazard ratio, CI: confidence infernal,

*the values had statistically significant differences *p*≤0,05.

### The tumor microenvironment based on VISTA and CD8+ TILs in patients with EOC

Given that VISTA protein expression was significantly correlated only with the density of CD8+TILs, we aimed to study the EOC immune microenvironment-based CD8+TILs and VISTA.

**Fig 3:**
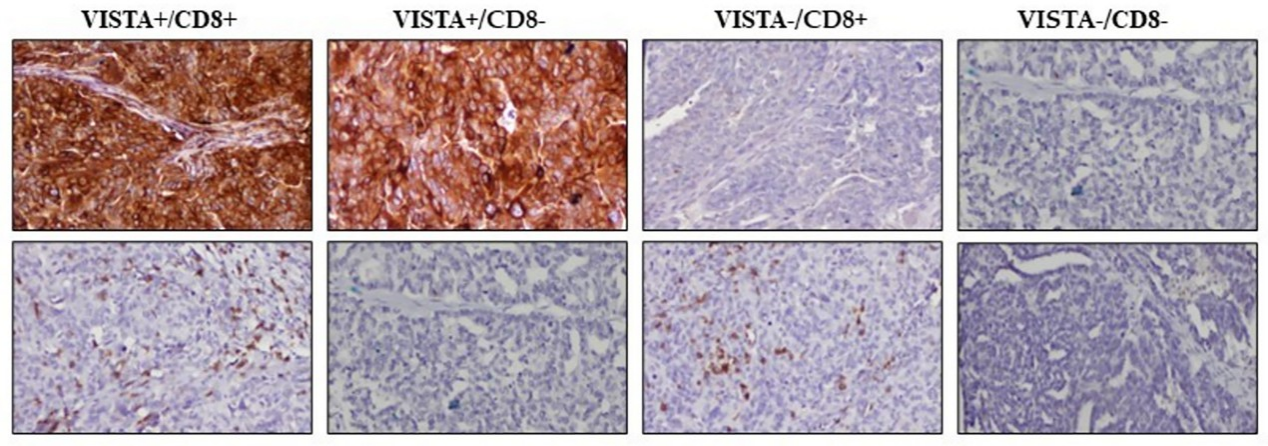
Classification of the different EOC immune microenvironment based on VISTA and CD8+TILs. Four immune subtypes based on VISTA and CD8+ immunohistochemical staining with VISTA+/CD8+, VISTA+/CD8-, VISTA-/CD8+, and VISTA-/CD8- (x200).

The CD8+TILs and VISTA immunohistochemistry analysis of the 126 EOC sections, allowed their classification on four immune subtypes; VISTA+/CD8+(64/126), VISTA+/CD8(31/126)-, VISTA-/CD8+(17/126) and VISTA-/CD8-(31/126) (Fig 3).

We subsequently assessed the relationship between the four immune subtypes and the prognosis of patients with EOC.

**Fig 4:**
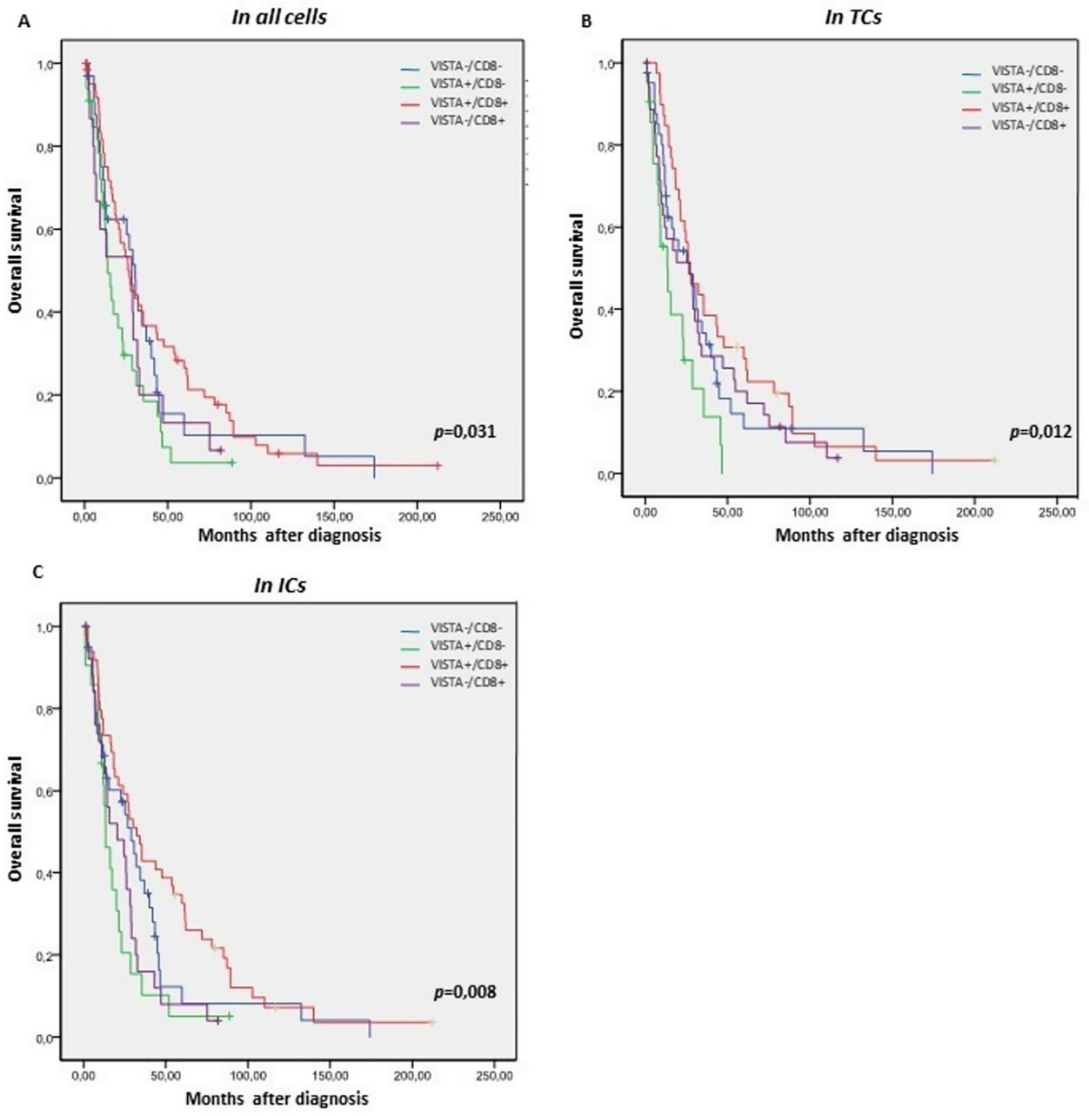
Kaplan-Meier curves of OS in the EOC according to the four immune subtypes based on VISTA and CD8+ expression. (A-C) Kaplan-Meier OS curves. (A) VISTA protein expression in all cells, (B) VISTA expression in TCs, (C) VISTA expression in ICs. The *p*-values of log-rank test are indicated in the figures.

Interestingly, using the Kaplan-Meier survival curves, we showed that patients with dual positive VISTA+/CD8+ subtypes had a better OS than the other subtypes. However, patients with VISTA+/CD8- had the worst OS in both TCs and ICs. It is worth noting that this correlation was more significant in ICs (*p* = 0,008) than in TCs (*p* = 0,012) (Fig 4).

### VISTA expression in IGRov1 cell line and PBMC of EOC patients

**Fig 5:**
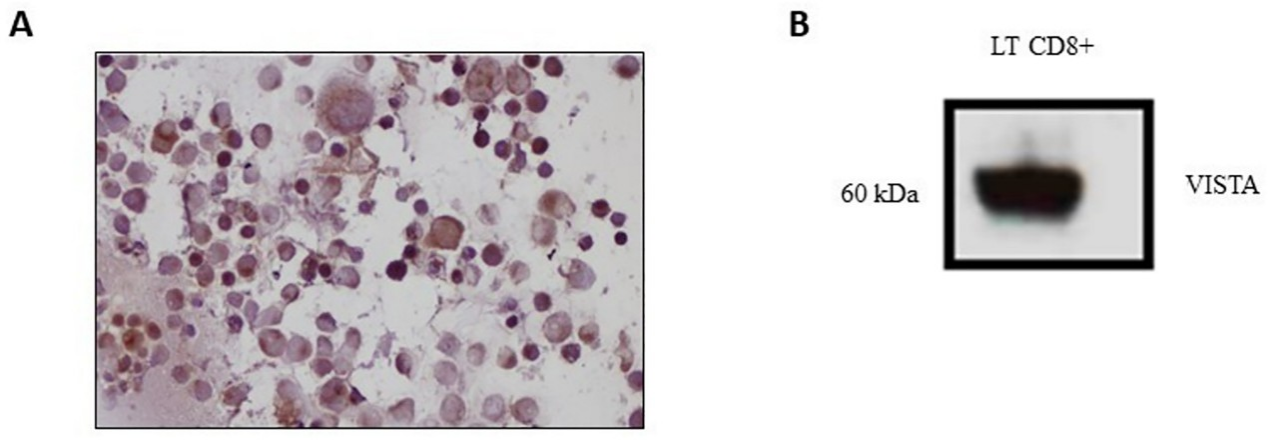
Protein expression of VISTA in EOC cells and in LT-CD8+ cells. A. Immunocytochemistry (ICC) in IGRov1. IGRov1 cells were cultured and collected. B. Western blot analysis. LTCD8+ cells isoleted from a patient with EOC and cultured, then proteins were extracted and analyzed using WB.

Immunocytochemistry showed a high VISTA protein expression in IGRov1 cells (Fig 5A).

Using western blot, VISTA was also detected in LT-CD8 cell lysates isolated from PBMC patient with EOC before undergoing chemotherapy (Fig 5B). This result confirms that VISTA is highly expressed in LT-CD8.

## Discussion

Recent data showed that conventional anti-tumor treatment (radiotherapy and chemotherapy) and anti-angiogenic drugs have positive immunological effects in stimulating antitumor responses and antagonizing the immune tolerance in EOC. However, despite this monitoring carried out by the immune system, more patients ultimately relapse, and their tumors eventually become chemotherapy resistant. The high recurrence rate is thought to be due to remaining drug-resistant cells,caused in part by cancer stem cells (CSC) [12, 13]. In this regard, ovarian stem cells could have a possible role in the EOC initiation and progression, by upregulating the pro-inflammatory cytokines during ovulation to induce a local microenvironment that will result in the normal ovarian epithelial cells transformation that may undergo an immunoediting process through a crosstalk between the infiltrating immune cells and ovarian stromal microenvironment toward the EOC progression [14].

Therefore, current research focuses on new anti-cancer drugs to improve the clinical response of EOC patients, such as immunotherapy by blocking the immune checkpoint VISTA.

The expression of VISTA and its prognostic impact has been studied in various malignancies, and it is in the first clinical trials. However, its expression in EOC was poorly studied in the literature [10].

Our data demonstrated that VISTA was expressed frequently in EOC patients’ tissues (64%), with comparable levels in TCs and ICs (42% *vs*. 48%). In addition, VISTA was also highly expressed in the Epithelial ovarian cancer IGRov1 cell line. In survival analysis, VISTA expression was not associated with OS, whereas dual positive VISTA+ /CD8+ presented a better OS. Our result is in accordance with other studies reporting that VISTA is expressed in 51% to 91% of EOC cases [2, 3]. Different studies showed a high-level of VISTA expression in different types such as endometrial cancer (100%), malignant pleural mesotheliomas (85%), lung cancer (99%) and gestational trophoblastic neoplasia (98,2%) [2, 11, 15, 16]. In contrast, lower expression was found in hepatocellular carcinoma (29,5%) and esophageal adenocarcinoma (22,2%) [3, 17].

VISTA expression has also been reported in gastric carcinomas (8,8%), hepatocellular carcinoma (16,4%), triple-negative breast carcinoma (TNBC) (18,5%), human non–small cell lung cancer (21%),pancreatic cancer cells (25,6%) [6, 16, 18–20]. Interestingly, in this study, we found that VISTA was significantly expressed in both TCs and ICs (42% *vs* 48%) as reported by other studies, which showed that in EOC, VISTA was observed in both tumor-infiltrating immune cells and tumor cells [3, 10], unlike the other tumors where it is more commonly expressed in tumor-infiltrating immune cells. The differential expression of VISTA may be related to the difference in immunogenicity among different types of cancer, as ovarian carcinoma is a highly immunogenic tumor characterized by the expression of this immune-negative checkpoint [21, 22].

According to immune editing, the high expression of VISTA in TCs may be due to decreased tumor immunogenicity to complete tumor proliferation and editing [21]. This is a strategy that the tumor cell uses to escape the immune system as Zong *et al*. proved that VISTA is highly correlated with the expression of genes involved in tumor escape [3].

The prognostic impact of VISTA status varies depending on the type of cancer. Interestingly, unlike other types of cancers, a significant decrease in VISTA expression was described in the advanced stages. VISTA expression in EOC is associated with advanced stages and higher metastatic risk, supporting the role of VISTA in tumor progression [6, 10].

In the literature, the expression of VISTA in ICs is correlated with a favorable prognosis in TNBC [19], esophageal adenocarcinoma [17], and hepatocellular carcinoma [6]. In contrast, VISTA positive ICs was not associated with OS in ovarian cancer and in oral squamous cell carcinoma [10, 23]. Zong et *al*. described a significant correlation between VISTA-positive TCs and OS in High-grade serous ovarian carcinoma [3].

Interestingly, we showed in the present study that VISTA expression in ICs was significantly associated with a high density of TILs, as assessed by univariate analysis.

More important, multivariate analysis showed a significant association between VISTA-positive staining and high density of CD8+ TILs only in ICs. Thus, the expression of VISTA promotes the recruitment of cytotoxic LTs in the TME, causing their inactivation, given its inhibitory role on LTs. Indeed, cytotoxic T lymphocytes (LT-CD8) supported by LT-CD4 (TH1) are potent effectors of tumor cell elimination [24]. Therefore, there may be a new strategy, that ovarian tumor cells use to escape the cytotoxic effect of LT-CD8+, which will be interesting to identify.

By studying the expression of VISTA and the level of CD8 + TILs in the TME, we found four immunological subtypes (VISTA+/ CD8+), (VISTA+/ CD8-), (VISTA+/ CD8+), (VISTA-/ CD8-), correlated with the overall survival of the patients. The dual-positive group (VISTA+/ CD8+) was associated with the best 5-year OS, while the immunosuppressive group (VISTA+/ CD8-) was associated with the worst one.

These results proved that the survival of patients was better when VISTA was expressed in the presence of a high density of CD8+. Our results were consistent with a previous study in other cancer (such as hepatocellular carcinoma), showing that VISTA expression was significantly associated with CD8+TILs [6]. Unlike TNBC, VISTA expression was highly correlated to CD4+ TILS. The dual VISTA+/ CD4+ subgroup showed favorable prognosis and VISTA-/ CD4- subtype was associated with a poor prognosis [19].

Recent *in vivo* studies showed that VISTA exerts a quiescent function on naive lymphocytes (LTs) and, therefore, their inactivation. Moreover, these effects were lost on specific LT under inflammatory conditions where the effect of VISTA was diminished or attenuated [25, 26].

Likewise, in the VISTA+/ CD8- subgroup, the TME is slightly or not inflammatory; VISTA suppresses the infiltrating LTs, leading to the lowest OS and the worst prognosis. Thus, VISTA correlates with a good prognosis when expressed with an elevated CD8+ count. Furthermore, in this study we observed a significant expression of VISTA by LT-CD8 in patient with EOC before undergoing chemotherapy. However, their alteration is weak in a highly inflammatory environment.

In this inflammatory TME, it is suggested that there is a molecule that targets VISTA and relieves its suppressive effect on cytotoxic lymphocytes (LT-CD8+) or that increases the pH of the extracellular medium to a basic pH which is unfavorable for the inhibitory action of VISTA on LT. In the literature, this inflammation could be explained in part by the obesity and metabolic diseases, such as the disregulation of PPARs (Peroxisome proliferator-activated receptors) that play a crucial role in macrophage infiltration, pro-inflammatory cytokines, inducing the inflammation, increasing the cancer risk and modulating pivotal cross-talk pathways for cell cycle, proliferation and differentiation [27, 28]. Further studies on CD8+/VISTA+ in TME are needed to better define the mode and conditions of action of this immuno-checkpoint.

Recent studies showed that VISTA might play a significant role in immunotherapy tolerance as its expression increased after CTLA4 blockade in prostate cancer [29] and after PD1 blockade in melanoma [30]. Therefore, our future study will explore the potential relationship between VISTA, PDL1/PD1, and CTLA4 in EOC and its synergistic anti-tumor effect.

Treatment with anti-VISTA mAb13F3 enhances cellular anti-tumor immunity in Blood cancer and melanoma [7] and hepatocellular carcinoma [6]. Furthermore, there are some clinical trials about this promising immuno-checkpoint, in a- phase- I trial (NCT02671955 and NCT02812875) in human progressed cancers and advanced solid tumors, respectively [19].

## Conclusions

The present study showed that VISTA expression was almost the same in ICs and CTs in EOC, with the same role in patient prognosis in terms of OS. To our knowledge, this is the first study, using a large series of EOC tumors, demonstrating that the expression of VISTA in TCs and ICs strongly correlates with the density of LT-C8+. We have also shown that VISTA+/CD8+ expression is an independent prognostic factor for the best 5-year OS. Given VISTA’s high frequency of expression in both tumor and tumor-associated inflammatory cells in these tumors, anti-VISTA therapies may be particularly effective in ovarian carcinoma. Despite these important findings, our study presented a few limitations, concerning the retrospective study and that the TME does not reflect the entire ovarian tumor given its intratumoral heterogeneity. Nevertheless, these data provide a relevant rationale for exploring VISTA as a novel immunotherapy target in EOC.

## Supporting information

S1 Graphical abstract (TIF)

S1 Raw images (PDF)
