# Supplementary figures and images for: VISTA+/CD8+ status correlates with favorable prognosis in Epithelial ovarian cancer
Source: PLoS One. 2023 Mar 23;18(3):e0278849. doi: 10.1371/journal.pone.0278849 (PMC10035885; doi:10.1371/journal.pone.0278849)

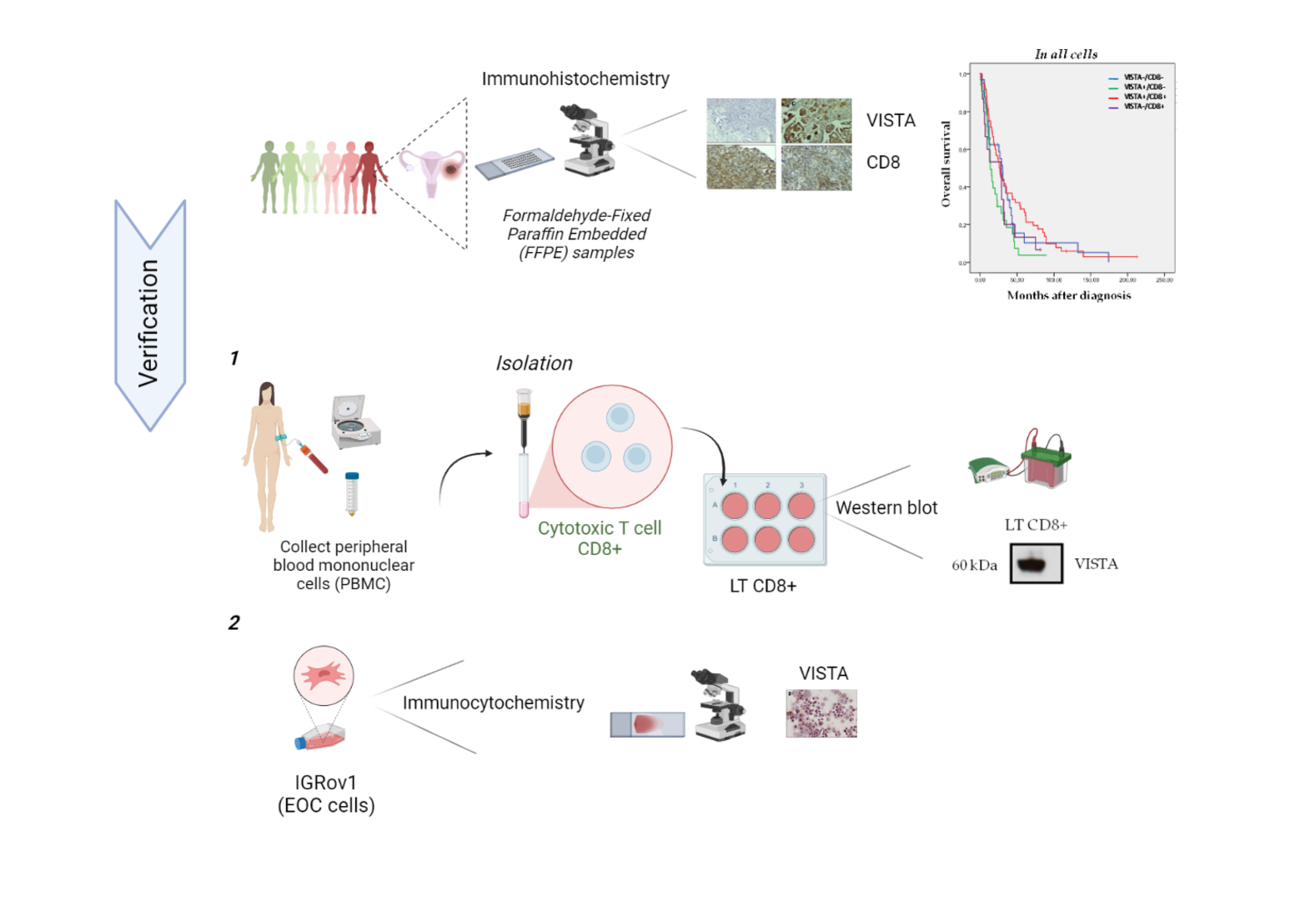

Supplement: S1 Graphical abstract — (TIF) [file pone.0278849.s001.tif]

$\beta$ -actin

VISTA

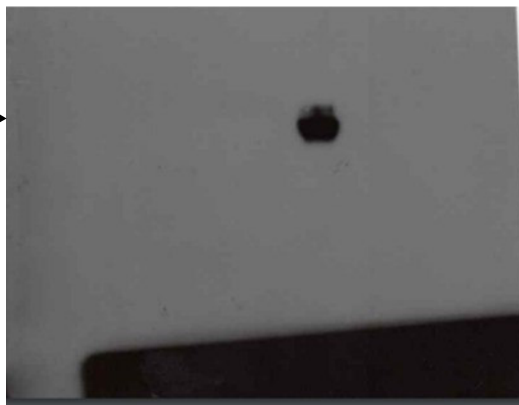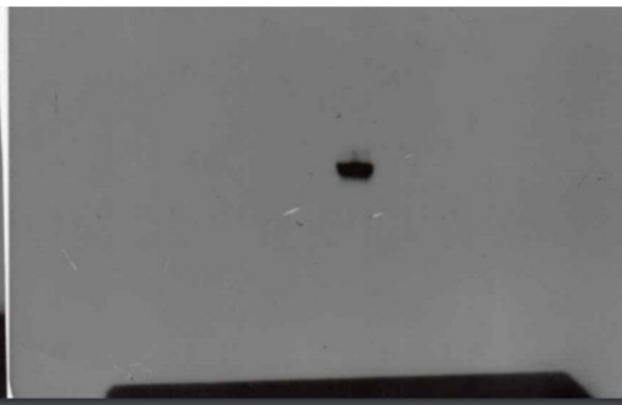

42 kDa →

← 60 kDa

10 seconds

60 seconds

Supplement: S1 Raw images — (PDF) [file pone.0278849.s002.pdf]
